# Enzymatic Hydrolysis-Derived Water-Soluble Carbohydrates from *Cacalia firma*: Evaluation of Antioxidant Properties

**DOI:** 10.3390/foods14081326

**Published:** 2025-04-11

**Authors:** Si-Young Ha, Hyeon-Cheol Kim, Jae-Kyung Yang

**Affiliations:** Department of Environmental Materials Science, Institute of Agriculture and Life Science, Gyeongsang National University, Jinju 52828, Republic of Korea; sy@gnu.ac.kr (S.-Y.H.); limjk19@naver.com (H.-C.K.)

**Keywords:** *Cacalia firma*, antioxidant, enzyme hydrolysis, carbohydrate, optimization

## Abstract

This research focused on producing water-soluble carbohydrates extracts from the leaves of the wild plant *Cacalia firma* using commercial enzymatic processes. Different enzymes and conditions were applied to the leaves to determine the optimal method for extracting carbohydrates. Enzymes used were Cellic CTec3 HS, Celluclast 1.5 L, Viscozyme L, Pectinex ultraSP-L, and Amylase AG. Pectinase, cellulase, and other enzymes are isolated from yeast, bacteria, or some higher plants and are commonly used to break down pectin, which is the cell wall or intercellular connective tissue in plant tissues, to soften fruit or vegetable tissues and to make sugars. They are commonly used to soften the tissues of fruits and vegetables, to produce sugars, or to increase the yield of juice in fruit processing. The resulting water-soluble carbohydrates demonstrated significant antioxidant capabilities in vitro, as evidenced by DPPH radical-scavenging and ABTS assays. Furthermore, the carbohydrates exhibited high levels of total polyphenol and flavonoid content. The extraction methodology was fine-tuned using response surface methodology alongside the Box–Behnken design, achieving a maximum carbohydrate yield of 129.7 mg/g, which was very close to the predicted value of 132.4 mg/g. The optimal conditions included an extraction temperature of 47.3 °C, a duration of 63 h, and a pH of 3.7 using Viscozyme L. This study offers a theoretical foundation for the development of natural carbohydrate antioxidants and lays the groundwork for large-scale production and utilization of *C. firma* leaf carbohydrates. These extracts, showing antioxidant activity, hold potential as functional ingredients in the food industry.

## 1. Introduction

Enzymatic carbohydrate production is one of the best methods. Enzymatic hydrolysis minimizes the desirable molecular weight and kinetic modifications of the end-product carbohydrate. There are two ways to produce carbohydrates: hydrolysis and synthesis, with hydrolysis currently being the most utilized method. There are enzymatic, chemical, and physical hydrolysis methods. Chemical hydrolysis produces monosaccharides and toxic furfural or HMF (hydroxymethylefurfural) and has the disadvantage of low yields. Physical hydrolysis methods are expensive and produce furfural and HMF. On the other hand, enzymatic hydrolysis has the advantages of high efficiency, specificity, and high yield. Since enzymes are substrate-selective, various biocatalysts need to be developed to produce high-quality bioactive carbohydrates.

Recent rises in national income have brought about shifts in dietary practices, with more people eating out and consuming processed foods, leading to issues of both excess and insufficient nutrition [1]. This has contributed to a rise in chronic conditions such as diabetes, obesity, hypertension, and hyperlipidemia. As a result, there is an increased emphasis on health, driving up the consumption of plant-based and functional foods known for their diverse physiological benefits [2]. These plant-based options are abundant in carbohydrates, including dietary fiber, which is increasingly recognized as a valuable functional ingredient due to its role in preventing and managing various health conditions [3]. Furthermore, dietary fiber is extensively employed in the food industry to improve the sensory qualities of food products, attributed to its unique physical characteristics [4].

*Cacalia firma*, belonging to the chrysanthemum family (Compositae), is a perennial plant scientifically known as *C. firma* (Komarov) Nakai [5]. This plant is native to Korea, flourishing in the shaded areas of dense mountain forests in the northern regions, specifically in Gyeonggi-do and Gangwon-do provinces. The plant features upright stems reaching 1–2 m in height, marked by distinctive white longitudinal stripes [6]. Numerous species within the Asteraceae family serve as fresh vegetables and greens, contributing to the well-documented presence of caffeoylquinic acid and terpenoid compounds in plants like balsam, chamomile, kudzu, and lettuce. While *C. firma* is known to possess a variety of bioactive compounds, its growth in mountainous areas limits its accessibility and general familiarity.

Mountain-grown wild greens are rich in proteins and bioactive compounds, including dietary fiber—an oleaginous polymeric compound—offering significant potential as functional food ingredients [7]. Despite their benefits, these compounds often exhibit low solubility, prompting the development of various extraction techniques [8]. Recently, enzyme-assisted biological extraction methods have gained attention [9]. This approach transforms insoluble polymers into water-soluble small molecules. Unlike the complex and potentially hazardous processes of physical and chemical hydrolysis, enzymatic methods offer substrate specificity, minimizing by-product formation and yielding a uniform digestate [10]. By adjusting factors such as substrate concentration, digestion time, temperature, and pH, specific digestates can be efficiently obtained. Consequently, numerous studies have explored the enzymatic degradation of cell walls to produce small-molecule, water-soluble extracts with functional properties [11]. For example, treating sesame porridge with cell-wall-degrading enzymes enhanced polyphenol content and antioxidant activity compared to untreated counterparts. Carbohydrates extracted from buckwheat husks using commercial enzymes like Viscozyme showed increased yields and were noted for their antioxidant, anti-inflammatory, and anticancer properties [12]. Additionally, enzymatically degraded soluble dietary fiber from buckwheat hulls using Celluclast and Viscozyme was found to inhibit glucose and bile acid absorption while exhibiting antioxidant effects [13].

This research focused on generating a water-soluble carbohydrate extract through enzymatic degradation, aiming to establish foundational data for using *C. firma* leaves as a functional food ingredient. This study involved selecting a cell-wall-degrading enzyme capable of enhancing hydrolysis efficiency and identifying the optimal conditions for hydrolysis with the chosen enzyme. Additionally, as a preliminary step to assess the functionality of the water-soluble carbohydrate extract produced under these optimal conditions, its antioxidant activity was evaluated.

## 2. Materials and Methods

### 2.1. Materials

Leaves of *C. firma* were sourced from a farm located in Cheongju-si, Chungcheongbuk-do, South Korea, and were promptly freeze-dried using a −80 °C freeze dryer (ILSHIN, Dongducheon-si, Republic of Korea) post-purchase. The freeze-dried leaves were then ground into a fine powder using a stainless steel grinder operating at 3000 W, 60 Hz, and 36,000 r/min. To ensure uniformity in particle size, the powdered leaves were sieved through a 40-mesh screen. For the study, five widely utilized cell wall hydrolases were selected: Cellic CTec3 HS, Celluclast 1.5 L, Viscozyme L, Pectinex ultraSP-L, and Amylase AG (Novozymes A/S, Bagsvaerd, Denmark). Details regarding the characteristics and activity ranges of these enzymes are provided in Table 1.

### 2.2. Enzyme Treatment of C. firma Using Various Types of Enzymes

To prepare the reaction mixture, 2 g of *C. firma* leaves were combined with 50 mL of citrate buffer, and each enzyme was introduced at a concentration of 40 units/mL. The mixture was then incubated in a shaking incubator at 100 rpm under specific conditions: pH 5.0, 50 °C, for a duration of 72 h. Following enzymatic treatment, the mixture was centrifuged at 4500 rpm for 10 min using a Union 32R Plus centrifuge (Hanil, Gimpo-si, Republic of Korea), resulting in the separation of an enzyme-treated solution and residue. The yield of the enzyme-treated residue was recorded, and the residue was stored at 4 °C. The enzyme-treated solution was subjected to heating in a 90 °C water bath for 10 min to deactivate the enzymes, followed by centrifugation at 2500 rpm for 10 min to collect the supernatant. This supernatant was subsequently utilized for the analysis of carbohydrate content.

### 2.3. Enzyme Treatment of C. firma Using Various Treatment Conditions of Enzymes

The enzyme treatment was conducted under varying conditions: a pH range of 3.0 to 6.0, temperatures between 40 °C and 60 °C, treatment durations from 24 to 72 h, and an enzyme concentration of 40 units/mL. Post-treatment, samples were centrifuged at 4500 rpm for 10 min using a Union 32R Plus centrifuge (Hanil, Gimpo-si, Republic of Korea), resulting in the separation of enzyme-treated liquid and residue. To deactivate the enzymes, the lysate was heated in a water bath at 90 °C for 10 min. Subsequently, the supernatant was isolated by centrifugation at 2500 rpm for 10 min. This supernatant, following enzyme inactivation, was then utilized for analyzing carbohydrate content.

### 2.4. Water-Soluble Carbohydrate Extraction

Carbohydrates were isolated from the enzyme-treated liquor based on the specific enzyme type and processing parameters. To facilitate this extraction, 98% alcohol was added at a ratio of 20:1 relative to the enzyme solution volume, and the mixture was incubated at 4 °C for 24 h. Following this, the mixture was centrifuged at 4500 rpm for 10 min using a Union 32R Plus centrifuge (Hanil, Gimpo-si, Republic of Korea) to remove the supernatant. The resulting precipitate was then subjected to freeze-drying at −80 °C for 72 h using a freeze dryer (ILSHIN, Dongducheon-si, Republic of Korea). The freeze-dried material was subsequently weighed to determine its carbohydrate content. We called this weight the carbohydrate yield.

### 2.5. Response Surface Methodology (RSM) Analysis for Carbohydrate Extraction from C. firma

A single factor test was conducted to determine the optimal enzyme treatment conditions—pH, temperature, and duration—for maximizing carbohydrate extraction from *C. firma*. This test served as a foundation for the RSM design by assessing the impact of each factor on carbohydrate yield and identifying the range that maximizes content. Viscozyme L was chosen for this test due to its superior carbohydrate extraction capability compared to other enzymes. The parameters tested included a pH range of 3.0 to 6.0, temperatures from 40 °C to 60 °C, treatment durations between 24 and 72 h, and an enzyme concentration of 40 units/mL. The RSM was developed using the Box–Behnken design, with statistical analysis performed using Design Expert software (version 13). Table 2 outlines the factor ranges for the 17 Box–Behnken design conditions, while Table 3 presents the carbohydrate yields under these conditions. From these 17 conditions, the optimal treatment settings with Viscozyme L were identified to achieve the highest carbohydrate content.

### 2.6. Bioactive Analysis of the Water-Soluble Carbohydrate Extracted Under Optimal Conditions

#### 2.6.1. Total Polyphenol Content Analysis

Polyphenolic compounds, including flavonoids, anthocyanins, tannins, catechins, isoflavones, lignans, and resveratrol, are prevalent throughout the plant kingdom and are abundant in fruits and leafy vegetables. Due to their numerous hydroxyl groups (-OH), polyphenols readily bind with various compounds, contributing to their potent antioxidant, anticancer, and anti-inflammatory properties. The total polyphenol content was assessed using a method outlined by Lee et al. [14], which involves the reaction of the Folin–Ciocalteu reagent with polyphenolic compounds to form a colored molybdenum blue complex. For the assay, 100 µL of the Folin–Ciocalteu solution was mixed with 100 µL of the extracted carbohydrate, and the mixture was allowed to react for 3 min at room temperature. Following this, 100 µL of 2% sodium carbonate was added, and the mixture was further reacted for 30 min at room temperature, with absorbance measured at 750 nm. The total polyphenol content was quantified using a standard curve created with gallic acid, and results were expressed as mg gallic acid equivalents (GAEs) per gram of sample.

#### 2.6.2. Flavonoid Content Analysis

The method outlined by Lee et al. [14] was used to determine the total flavonoid content. In this assay, 100 µL of the extracted carbohydrate solution (1 g/50 mL methanol) was combined with 100 µL of a 2% aluminum chloride solution in methanol. This mixture was allowed to react at room temperature for 10 min. Absorbance was then measured at 430 nm. The total flavonoid content was calculated using a standard curve generated with quercetin, and results were expressed as mg quercetin equivalents (QE) per gram of sample.

#### 2.6.3. DPPH Radical-Scavenging Activity Analysis

The DPPH antioxidant activity was evaluated following the procedure detailed by Lee et al. [14]. In this assay, 150 µL of DPPH reagent, prepared in ethanol at a concentration of 150 µM, was mixed with 100 µL of the extracted carbohydrate solution (1 g/50 mL methanol). The reaction was allowed to proceed for 30 min at room temperature, protected from light. Absorbance was recorded at 517 nm using a UV-spectrophotometer, with ascorbic acid serving as the positive control for comparison.

#### 2.6.4. ABTS Antioxidant Activity Analysis

The ABTS antioxidant activity was measured following the method outlined by Lee et al. [14]. The ABTS solution was prepared by combining 7 mM of ABTS with 2.45 mM of potassium persulfate and allowing the mixture to react in the dark for 24 h at room temperature. This solution was then diluted with methanol to achieve an absorbance of 0.7 ± 0.02 at 735 nm. For the assay, 190 µL of the ABTS solution was mixed with 10 µL of the extracted carbohydrate (1 g/50 mL methanol), and the reaction was carried out for 6 min at room temperature. Absorbance was recorded at 517 nm using a UV-spectrophotometer, with ascorbic acid used as a positive control.

### 2.7. Statistical Analysis

Statistical analyses were performed using SAS program (9.4M8 version; SAS Institute, NC, USA), and one-way ANOVA test was conducted to determine statistical significance at the *p* < 0.05 level.

## 3. Results

### 3.1. Carbohydrate Content of Enzyme Treated C. firma as Various Enzymes Types

**Figure 1:**
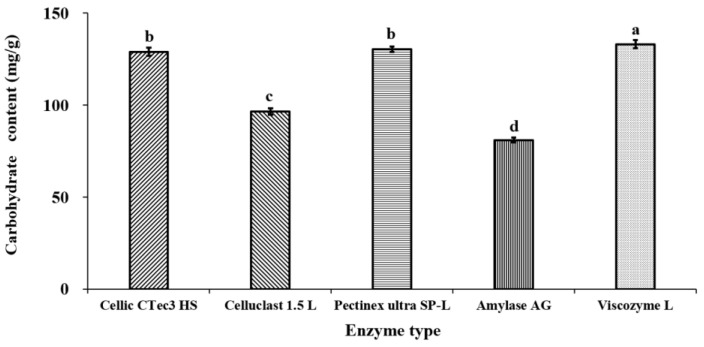
Carbohydrate content of *C. firma* according as enzyme type. Different lowercase letters indicate statistically significant differences (*p* < 0.05).

The carbohydrate content in enzyme-treated *C. firma* leaves varied depending on the enzyme used, with values ranging from 81 mg/g to 133 mg/g. Among the enzymes tested, the highest carbohydrate content was observed with Viscozyme L, followed by Pectinex ultra SP-L, Cellic CTec3 HS, Celluclast 1.5 L, and Amylase AG, in that order (Figure 1). Enzymatic extraction facilitates the transformation of insoluble polymeric compounds into water-soluble small molecules. By modifying parameters such as substrate concentration, digestion time, temperature, and pH, it is possible to optimize the digestate to contain a high concentration of the desired substance. Consequently, assessing the impact of varying conditions like pH during enzymatic treatment on carbohydrate content is essential.

### 3.2. Effect of Enzyme Treatment Conditions on Carbohydrate Content of Enzyme Treated C. firma

**Figure 2:**
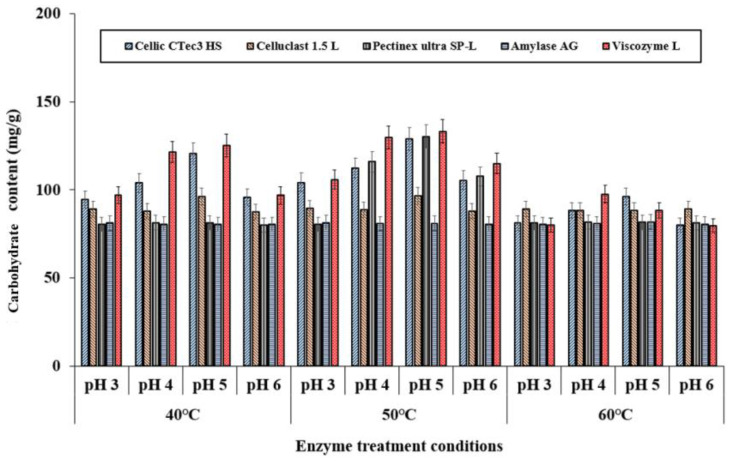
Carbohydrate content of *C. firma* according as enzyme treatment conditions. The data indicate averages ± standard deviation.

**Figure 3:**
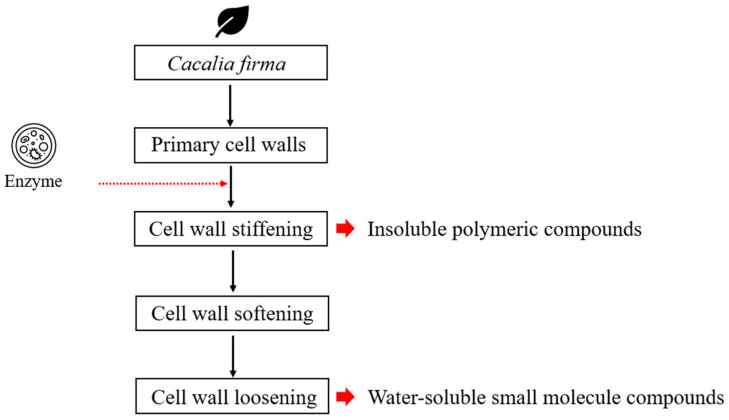
The positive impact of enzymatic treatment on the extraction of large amounts of carbohydrates.

Carbohydrate content in enzyme-treated folding-screen leaves varied between 79 mg/g and 133 mg/g (Figure 2). Viscozyme L resulted in the highest carbohydrate yield, particularly under conditions of a 72 h reaction at 50 °C in a buffer with a pH of 5. This increase in carbohydrate content indicates that *C. firma* leaves are rich in proteins and bioactive compounds, such as dietary fiber. The enzymatic treatment proved effective for extracting these bioactive substances, likely due to their low solubility (Figure 3).

### 3.3. Optimization of Enzyme Treatment Conditions for Maximum Carbohydrate Content of C. firma

**Figure 4:**
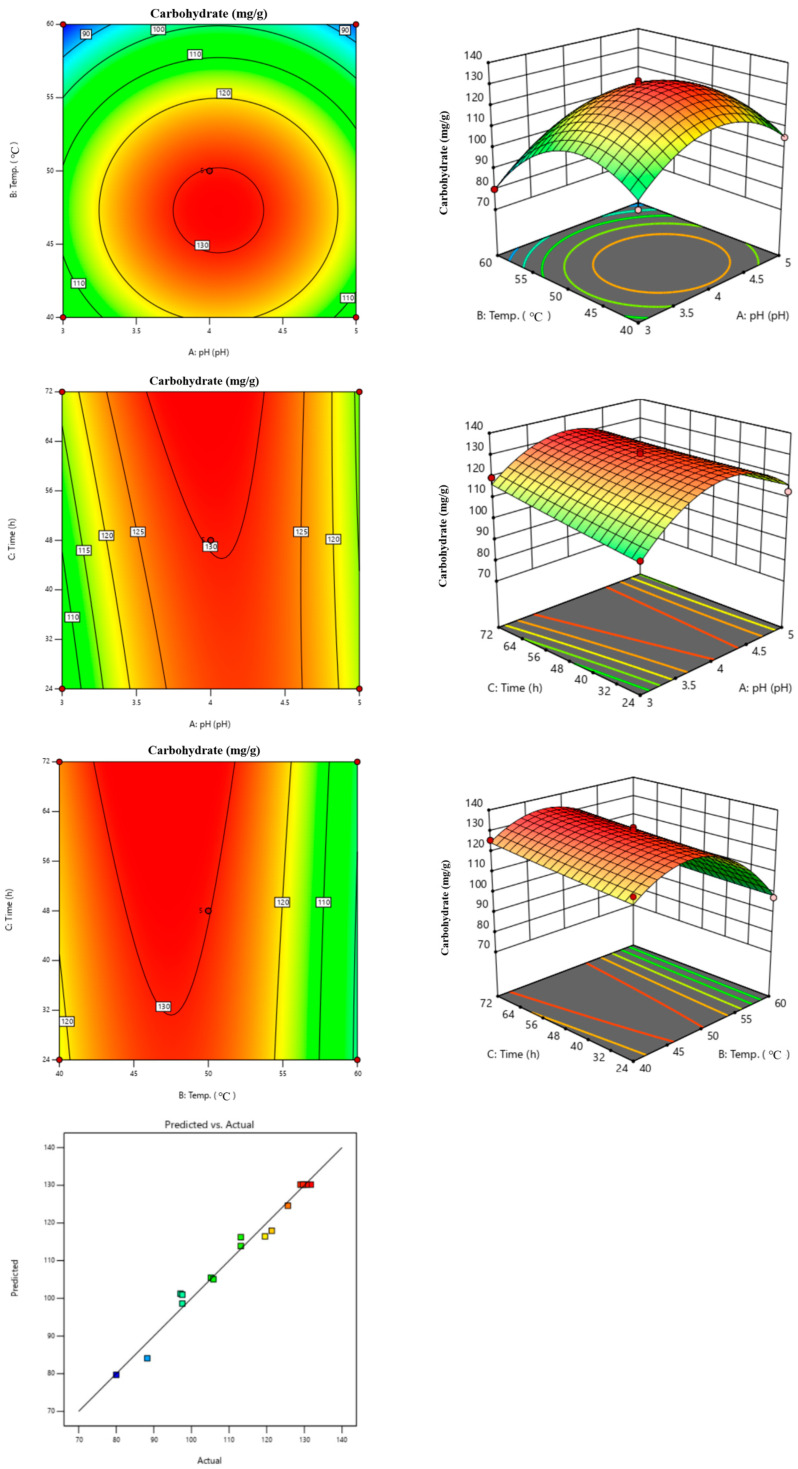
Three-dimensional plot and actual vs. predicted graph.

ANOVA statistical analysis was conducted to assess the reliability of the 3D plots generated, with results presented in Table 4 and Figure 4. The analysis indicated that among the three variables—pH, treatment temperature, and treatment time—treatment temperature had a significant impact on carbohydrate content, with a *p*-value of <0.0001, as evidenced by the 3D plot curves. The ANOVA confirmed that the model was reliable, with an R^2^ value of 0.9802. We used this model to derive regression equations to make predictions about carbohydrates. We need to conduct future research to propose regression equations using only the influential factors. In the context of ANOVA, ‘Lack of Fit’ suggests that the polynomial regression model may not adequately describe the response if the variation it causes is significant. However, in this study, the Lack of Fit had a *p*-value of 0.0854, indicating that the model is indeed reliable.

The 3D plot revealed that the treatment temperature had the steepest slope, indicating it as the most influential factor on carbohydrate extraction variables. The plot illustrates the impact on carbohydrate content, where a gentle slope suggests a minor effect, and a steep slope signifies a major impact. Consequently, the enzyme treatment conditions affecting the carbohydrate content of folding sesame leaves were ranked in order of influence as follows: treatment temperature > pH > treatment time. We established pH, temperature, and time as factors for optimal carbohydrate extraction conditions. As a result, we found that temperature as a single factor had the greatest influence on carbohydrate extraction, and pH and temperature as square root factors had the greatest influence, as shown in Table 4. From these results, we suggest that enzyme treatment time is relatively ineffective in determining carbohydrate content. Enzymes are highly active at specific pH and temperatures. At temperatures too low, the enzyme’s action slows down, and at temperatures too high, the enzyme’s protein structure is deformed and unable to function properly. Above the optimum temperature, the protein is denatured by heat and cannot catalyze. Enzymes denatured at high temperatures cannot regain activity when the temperature is lowered again. We propose that the reactivity of these enzymes under specific conditions is responsible for the variation in the extraction yield of carbohydrates.

Figure 4 illustrates the model’s reliability, showing that the predicted and actual values align closely along the trend line within a specified range. Utilizing the response surface methodology results, the carbohydrate content in enzyme-treated folding-screen leaves can be estimated based on variable values, with the predictive equation provided in Equation (1) below:

Carbohydrate content, mg/g = −645.90726 + 149.50045A + 19.09029B + 0.837447C + 0.003794AB − 0.142878AC − 0.004477BC − 17.58499A^2^ − 0.199637B^2^ + 0.000539C^2^ (A: pH; B: Treatment temperature, °C; C: Treatment time, h) (1)

**Figure 5:**
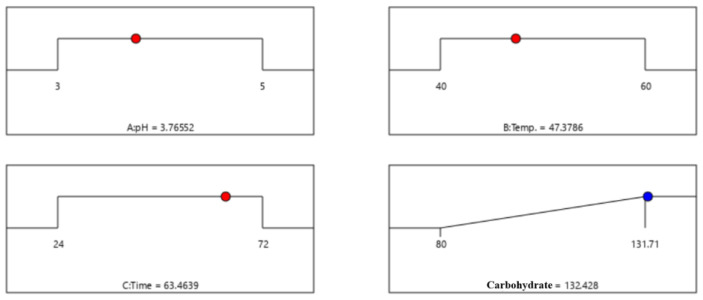
Optimized enzyme treatment conditions for carbohydrate extract from *C. firma*. The red dots represent the optimal conditions for each factor, and the blue dots represent the highest value of carbohydrat under optimal conditions.

Figure 5 presents the ideal enzymatic treatment parameters for achieving the highest carbohydrate yield. In response, surface calculations were used to determine the optimal conditions. The optimal conditions identified were a pH of 3.7, a temperature of 47.3 °C, and a treatment duration of 63 h. Under these conditions, the extraction of carbohydrates from *C. firma* leaves reached a peak content of 132.4 mg/g. Using these predicted conditions, the actual measurement at the laboratory scale analyzed 141 mg/g of carbohydrate. We repeated this measurement three times and concluded that the predicted and measured values were similar.

### 3.4. Total Polyphenol Content of Carbohydrate Extracted from C. firma

**Figure 6:**
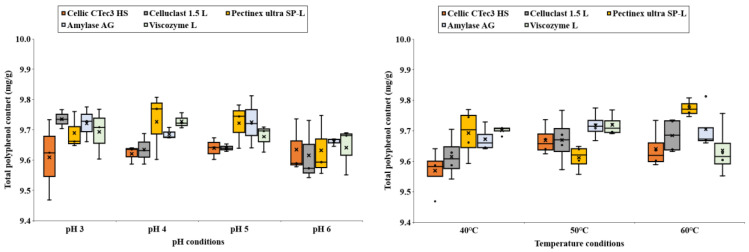
Polyphenol content of carbohydrate extracted from *C. firma*.

The polyphenol content in enzyme-treated *C. firma* leaves varied based on the conditions of enzyme treatment (Figure 6). Phenolic compounds are known to associate with cell wall carbohydrates, which form a complex structure comprising 30% neutral carbohydrates (such as cellulose, xyloglucan, arabinan, galactan, xylan, and mannan), 20% acidic pectic substances, 15% insoluble proanthocyanidins, lignin, structural proteins, and phenols [15]. These carbohydrates include hydrogen groups and aromatic and glycosidic oxygen atoms capable of forming hydrogen bonds and hydrophobic interactions with polyphenols [16]. Thus, it is suggested that enzymatic degradation of pectin and other cell wall components facilitated the release of phenolic compounds. Box plots are a popular visualization method used during data analysis. Box plots allow you to see the mean, standard deviation, and variance, and make it easy to compare different data sets. It can represent data with five different statistics, which are separated by points or lines: minimum, maximum, upper quartile, median, and lower quartile. We used this data to represent the range over which the conditions of enzymatic processing can affect polyphenol content.

### 3.5. Flavonoid Content of Carbohydrate Extracted from C. firma

**Figure 7:**
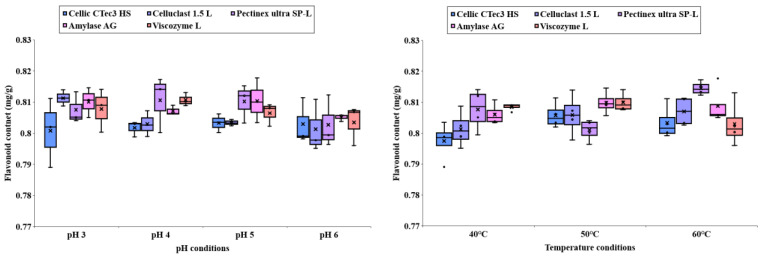
Flavonoid content of carbohydrate extracted from *C. firma*.

Figure 7 shows how the flavonoid content in *C. firma* leaves changes with different enzymatic treatment conditions. There is a growing interest in harnessing biologically active compounds, including flavonoids and phenolic acids, from processed plant residues. This interest has driven the use of hydrolytic enzymes targeting plant cell walls [17]. In a boxplot, the median is the most important number, meaning that 50% of the data is distributed at the top and the other 50% at the bottom. This data shows that flavonoid content is affected by enzyme type, processing temperature, and processing pH. This result suggests that it is important to choose enzyme processing conditions to determine flavonoid content.

### 3.6. Antioxidant Activity (DPPH Radical-Scavenging Activity and ABTS Antioxidant Activity) of Carbohydrate Extracted from C. firma

**Figure 8:**
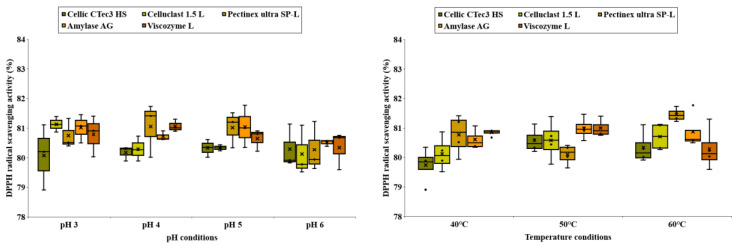
DPPH radical-scavenging activity of carbohydrate extracted from *C. firma*.

**Figure 9:**
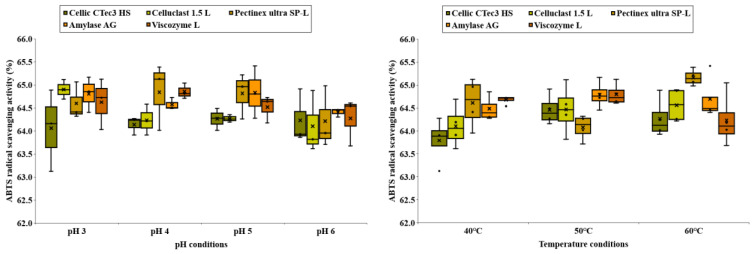
ABTS antioxidant activity of carbohydrate extracted from *C. firma*.

Figure 8 and Figure 9 depict the DPPH and ABTS antioxidant activities of *C. firma* leaves under various enzymatic treatment conditions. Enzymes that facilitate the release of polyphenols work by catalyzing the hydrolysis of ester and depside bonds found in hydrolyzable tannins or gallic acid esters, like epigallocatechin O-gallate or epicatechin O-gallate, resulting in the liberation of gallic acid or glucose [18]. Utilizing enzymes that promote the release of gallic acid from plants can address issues such as the browning caused by tannic acid [19]. Gallic acid, released through enzymatic treatment, is recognized for its potent antioxidant effects [20]. This study also indicated a positive correlation between polyphenol content and antioxidant activity. The average or standard deviation that we typically use to represent data has the potential to convey a skewed meaning when there are outliers in the data. Boxplots make it easy to determine how much outliers are included. A boxplot of antioxidant activity by enzyme treatment conditions reveals that different enzyme types have different conditions for activity. Since we used a variety of enzymes in this paper, we hypothesized that the enzyme treatment process plays a role in determining the antioxidant activity. This hypothesis is confirmed by the data shown in Figure 8 and Figure 9.

**Figure 10:**
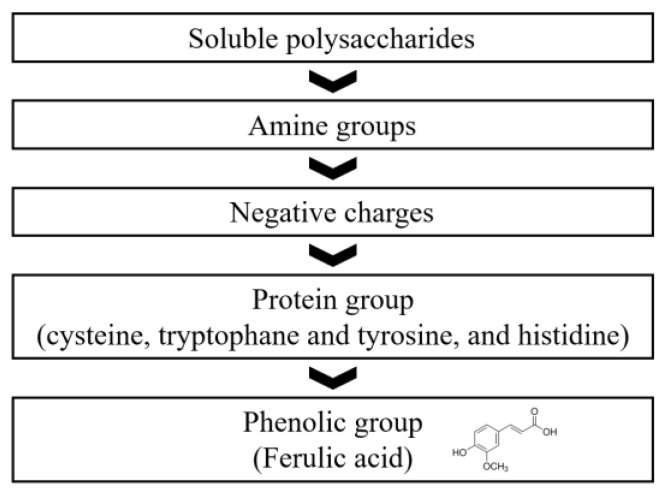
Proposed principles of key properties associated with the antioxidant properties of carbohydrates.

An important deconstruction of the antioxidant properties of carbohydrates is presented here. Regardless of their origin, carbohydrates can scavenge radicals or inhibit the formation of radicals through metal chelation. A survey of the literature has shown that the in vitro chemical antioxidant activity of carbohydrates is highly dependent on their solubility, sugar ring structure, molecular weight, occurrence of positively or negatively charged groups, protein portion, and covalently attached phenolic compounds. The last two structural features may contribute more to carbohydrate antioxidant activity, scavenging DPPH-, ABTS-+, and exhibiting metal-reducing properties that are typically absent in pure carbohydrate fractions free of phenols and proteins (Figure 10).

## 4. Discussion

Enzymes like pectinases, cellulases, hemicellulases, and glucanases have been shown to effectively release complex polyphenols from cell walls, aiding in the liberation of nutrients that are otherwise confined within the cell wall matrix [21]. Previous research has demonstrated that enzymatic processes can enhance flavonoid activity [22], and our findings also indicate that enzymatic treatment of *C. firma* leaves may promote the release of flavonoids.

Investigating the enzymatic treatment of *C. firma* is crucial for recovering phenols and producing extracts that can serve as food supplements or innovative functional ingredients. By hydrolyzing complex carbohydrates and polyphenols into simpler sugars and phenolic compounds, the availability of biologically active substances can be enhanced. The enzymes utilized in this study proved effective in increasing these bioactive compounds. It is known that monomeric and some oligomeric polyphenols are easily absorbed [23], while polymeric forms are less so. Thus, understanding the impact of enzymatic treatment on the polyphenol content and antioxidant activity of psyllium seeds provides valuable data for leveraging wild plants. Among the treatments, Viscozyme L demonstrated the highest bioactivity, followed by the enzyme solution treated with Pectinex ultra SP-L.

This study successfully optimized the extraction process for *C. firma* carbohydrates to enhance yield. Using the BBD experiment and RSM analysis, the optimal conditions identified were an extraction temperature of 47.3 °C, a duration of 63 h, and a pH of 3.7 with Viscozyme L. Under these conditions, the experimental yield of *C. firma* carbohydrates was 129.7 mg/g, closely matching the predicted yield of 132.4 mg/g. Additionally, the carbohydrates demonstrated significant antioxidant capabilities, as shown by their DPPH radical-scavenging and ABTS antioxidant activities, suggesting their potential as a novel natural antioxidant for functional foods or feed additives. Future research should focus on purification, structural analysis, and functional evaluation of *C. firma* carbohydrates to expedite its development into an effective product. This study provides a theoretical foundation for further systematic exploration and the rational development and use of *C. firma* carbohydrates.

The information reviewed here emphasizes the relevance of establishing enzymatic processing relationships between the possible antioxidant properties of carbohydrates and their use in food, pharmaceutical, and materials science. To achieve this, future research should follow a strategy that fully mitigates the bias of other compounds on carbohydrate antioxidant properties, which should include using water solubility to remove polyphenols to prevent them from occurring as contaminants during carbohydrate extraction. Enzymatic treatment strategies may pave the way for the development of new carbohydrate design methodologies that can improve antioxidant effects over the currently classical antioxidant active compounds. Until then, despite falling under the antioxidant concept given their tendency to inhibit oxidative reactions, the naming of carbohydrates as antioxidants should be applied with caution and only in well-established contexts, taking into account their chemical properties. This will allow for better use and development of strategies to use carbohydrates in biomedical applications, cosmetics, food packaging and preservation, and food supplements.

## Figures and Tables

**Table 1:** Characteristics and activity of various cell-wall-degrading enzymes used in the study.

Enzyme	Characteristics			
	Sources	Optimal pH	Optimal Temperature (°C)	Enzyme Activity Target
Cellic CTec3 HS	*Trichoderma reesei*	4.7–5.2	50–55	Cellulase, β-glucosidases, hemicellulase
Celluclast 1.5 L	*Trichoderma reesei*	4.5–6.0	50–60	Cellulase
Viscozyme L	*Aspergillus aculeatus*	3.5–5.5	25–55	Arabanase, cellulase, β-glucanase, hemicellulose, xylanase
Pectinex ultraSP-L	*Aspergillus aculeatus*	4.0–5.0	55–60	Polygalacturonase
Amylase AG	*Aspergillus niger*	4.5	60	Glucoamylase

**Table 2:** Single factor range for RSM Box–Behnken design.

Factor	Code		
	−1	0	1
pH	3.0	4.0	5.0
Treatment temperature (°C)	30	50	60
Treatment time (h)	24	48	72

**Table 3:** Seventeen designs of RSM analysis for carbohydrate extraction from *C. firma*.

Run	pH	Treatment Temperature, °C	Treatment Time, h	Carbohydrate Content, mg/g
1	5	40	48	105.18
2	3	60	48	80.00
3	3	50	72	119.54
4	4	50	48	129.62
5	3	50	24	105.83
6	4	50	48	128.99
7	3	40	48	97.09
8	4	60	24	97.57
9	4	50	48	131.71
10	5	50	72	113.12
11	4	50	48	130.59
12	4	40	24	121.33
13	4	40	72	125.63
14	4	50	48	129.98
15	5	50	24	113.12
16	4	60	72	97.57
17	5	60	48	88.25

**Table 4:** Analysis of variance of optimization models for improved carbohydrate content.

Source	F-Value	*p*-Value	
Model	38.54	<0.0001	Significant
A	3.05	0.1240	
B	75.93	<0.0001	
C	3.34	0.1102	
AB	0.0005	0.9832	
AC	3.88	0.0896	
BC	0.3805	0.5568	
A^2^	107.31	<0.0001	
B^2^	138.31	<0.0001	
C^2^	0.0334	0.8601	
Lack of fit	4.67	0.0854	Not significant
R^2^ = 0.9802			

A: pH. B: treatment temperature, °C. C: treatment time, h.

## Data Availability

The original contributions presented in the study are included in the article, further inquiries can be directed to the corresponding author.
